# On-demand plerixafor added to high-dose cyclophosphamide and pegylated recombinant human granulocyte colony-stimulating factor in the mobilization of patients with multiple myeloma: a treatment with high effectiveness, convenient, and affordable cost

**DOI:** 10.3389/fonc.2023.1306367

**Published:** 2024-01-17

**Authors:** Li-qiong Hou, Jun-Ru Liu, Jing-Li Gu, Mei-Lan Chen, Li-Fen Kuang, Bei-Hui Huang, Wai-yi Zou, Juan Li

**Affiliations:** Department of Hematology, The First Affiliated Hospital of Sun Yat-sen University, Guangzhou, China

**Keywords:** pegylated granulocyte colony-stimulating factor, cyclophosphamide, plerixafor, stem cell mobilization, multiple myeloma

## Abstract

**Objective:**

The combination of high-dose cyclophosphamide (HD-Cy) (3g/m^2^) plus granulocyte colony-stimulating factor (G-CSF) and on-demand plerixafor (PXF) has been considered an effective mobilization regimen of patients with multiple myeloma(MM). However, the daily multi-injection regimen of G-CSF poses challenges. This study delves into the efficiency and cost implications of a novel approach, using HD-Cy alongside pegylated G-CSF (PEG G-CSF) and on-demand PXF. Unlike G-CSF, which necessitates daily injections, the half-life of PEG G-CSF extended allows for a single injection.

**Methods:**

A retrospective analysis was conducted on 350 MM patients, which were categorized based on their mobilization regimens: Cy+PEG G-CSF+/-PXF (n=66), Cy+PEG G-CSF (n=91), Cy+ G-CSF (n=169), and G-CSF+PXF (n=24).

**Results:**

Mobilization with Cy+PEG G-CSF+/-PXF(8.79)yielded a notably higher median CD34^+^ cell count compared to the other regimens: Cy+PEG G-CSF(4.96), Cy+G-CSF (4.65), and G-CSF+PXF (2.99) (P<0.001). The percentage of patients who achieved >6×10^6^/kg CD34+ cells was significantly higher in the Cy+PEG G-CSF+/-PXF group (77.3%) than in the other mobilization regimens: Cy+PEG G-CSF (41.8%), Cy+ G-CSF (37.3%), and G-CSF+PXF (8.3%) (P<0.001). From a cost perspective, the Cy+PEG G-CSF+/-PXF approach was more economical than the G-CSF+PXF strategy but was marginally costlier than the other two methods. A multivariate assessment highlighted that the combination of Cy+PEG G-CSF with on-demand PXF had a superior potential to achieve the desired harvest (6×10^6^/kg) compared to the Cy+PEG G-CSF protocol without PXF. The incremental cost-effectiveness ratio for each 1% increase in the probability of achieving a successful optimal harvest was $ 97.02 per patient. The incidence of neutropenic fever was 3.0% in the Cy+PEG G-CSF+/-PXF group.

**Conclusion:**

The combination of on-demand PXF with HD-Cy and PEG G-CSF offers a cost-effective approach with a high mobilization success rate, manageable side effects, and the convenience of fewer injections. It stands as a promising mobilization strategy for MM patients.

## Introduction

In the current landscape of novel treatments, autologous stem cell transplantation (ASCT) consistently plays a crucial role for eligible newly diagnosed multiple myeloma (MM) patients (1, 2). The advantage of tandem ASCT, especially for those at high risk, underscores the need for an optimal collection of hematopoietic stem cells (HSCs) (3, 4). Additionally, salvage ASCT has shown the potential to enhance overall survival for MM patients experiencing a relapse after an initial ASCT (5). It is important to collect enough stem cells for two or three transplants at the first mobilization. The success of ASCT largely rests on acquiring a significant number of CD34^+^ cells, ensuring rapid and long-lasting engraftment. While the minimum requirement for CD34^+^ cells stands at 2 × 10^6^/kg for a single ASCT and 4 × 10^6^/kg for tandem ASCT, many consider the safer benchmarks to be 3 and 6 × 10^6^/kg, respectively (6, 7). For younger patients, aiming for a higher count becomes imperative, anticipating potential needs during relapse. The International Myeloma Working Group (IMWG) recommends a minimum target of 4 × 10^6^ CD34^+^ cells/kg. However, ideally, securing an average of 8-10×10^6^ CD34^+^ cells/kg ensures most MM patients are equipped for two ASCTs during their treatment journey (8).

Effective and economical collection of ample HSCs, with minimal complications and fewer apheresis sessions, is the ultimate objective of mobilization. Currently, granulocyte-colony stimulating factor (G-CSF) is a common method, either standalone or paired with chemotherapy, for HSC collection. Yet, G-CSF monotherapy mobilization often faces a high failure rate, identified by a yield falling below 2 × 10^6^ CD34^+^/kg (9). One previous study indicated that a mere 34% of MM patients relying on G-CSF-only mobilization achieved the target of ≥ 6 × 10^6^/kg CD34^+^ cells (10). Adding high-dose cyclophosphamide (HD-Cy) to G-CSF does enhance HSC yield (11), but about 30% of patients still struggle to secure more than 4 × 10^6^/kg CD34^+^ stem cells (12). Regrettably, a smaller fraction even hits the 6 or 8 × 10^6^/kg mark, which is insufficient for two transplants. Failed initial mobilizations often necessitate a subsequent mobilization using backup regimens. However, these can lead to a higher rate of mobilization failure, heightened toxicity, morbidity, and costs (13–15). Consequently, patients who can not mobilize effectively miss out on ASCT and adequate treatment. Plerixafor (PXF), a CXCR4 antagonist, is an effective mobilizing agent. However, the cost of PXF is high. If universally adopted in initial MM patient mobilizations, the costs related to HSC harvesting would surge (16). The strategy of administering PXF ‘on-demand’, following an initial mobilization using either standalone G-CSF or a combination of chemotherapy and G-CSF, targets those patients displaying early indicators of inadequate mobilization. By reserving PXF for these specific cases, potential healthcare costs can be curtailed, making this approach increasingly favored in contemporary medical practice (17, 18). Research suggests that the mobilization efficiency of the combined regimen of Cy, G-CSF, and on-demand PXF surpasses that of the combination of G-CSF and on-demand PXF (19–21). However, the necessity for daily, multiple injections with G-CSF poses a challenge. Pegylated granulocyte colony-stimulating factor (PEG G-CSF) is a pegylated form of G-CSF. Its enhanced half-life (33 vs. 3-4 h) translates to the convenience of a single dose administration (22, 23). Multiple investigations affirm the efficacy of PEG G-CSF in ensuring an optimal HSC yield for ASCT. Notably, these studies suggest that PEG G-CSF not only presents a manageable side-effect profile but also outperforms standard G-CSF in cost-effectiveness and patient convenience (24–27). In addition, Luciano et al. highlighted that a solitary 12 mg dose of PEG G-CSF exhibited superior HSC mobilization capabilities compared to G-CSF. This not only streamlined the mobilization process but also reduced the dependency on PXF for patients diagnosed with either multiple myeloma or lymphoma (25).

Therefore, we postulate that combining HD-Cy with PEG G-CSF and on-demand PXF can enhance the efficiency and convenience of HSC mobilization while curtailing associated costs. Given the current paucity of prospective study on this topic, we offer a retrospective study, drawing from real-world data. Specifically, we contrast the outcomes of four distinct mobilization regimens: Cy+PEG G-CSF+/-PXF, Cy+PEG G-CSF, Cy+G-CSF, and G-CSF+PXF, all administered to MM patients poised for ASCT.

## Methods

### Patients

This retrospective study compared the use of four different mobilizing strategies in patients with MM eligible for ASCT at The First Affiliated Hospital of Sun Yat-sen University: Cy+PEG G-CSF+/-PXF (n=66) versus Cy+PEG G-CSF (n=91) versus Cy+ G-CSF (n=169) versus G-CSF+PXF (n=24). The analysis included patients with MM eligible for ASCT who underwent the first stem cell mobilization attempt (those with a second mobilization for salvage ASCT were excluded) between August 2008 and March 2023. The eligibility criteria included the following: (i) diagnosed with MM as defined by the International Myeloma Working Groups after induction treatment; (ii) 18–70 years old; (iii) Eastern Cooperative Oncology Group (ECOG) performance status ≤ 3; (iv) normal kidney and live function; and (v) no history of cardiac arrhythmias, congestive heart failure, or severe coronary artery disease. This study was conducted according to the Declaration of Helsinki and approved by the institutional review board of First Affiliated Hospital of Sun Yat-sen University.

### Mobilization and apheresis

Four groups of patients received the following mobilization regimens:

Cy+G-CSF: Patients received Cy 3 g/m^2^ and on the next day, they began G-CSF 300 μg/d, which was continued until collection was complete. Cy+ PEG G-CSF: Patients received Cy 3 g/m^2^ and one dose of PEG G-CSF 6 mg was administered 24 hours after Cy. Cy+PEG G-CSF+/-PXF: Patients received Cy 3 g/m^2^ and one dose of PEG G-CSF 6 mg was administered 24 hours after Cy. On-demand plerixafor (20 mg or 0.16 mg/kg in case of reduced renal function) was reserved for patients with an absolute CD34+ cell count of <20 cells/uL before apheresis or patients with a Day 1 CD34+ yield of < 3×10^6^/kg CD34+ cells. We administered mesna, hydration, alkalization of urine, and diuretic to each patient during CTX mobilization to prevent hemorrhagic cystitis. Daily monitoring of CD34+ cells in peripheral blood was performed by flow cytometry, starting from day +8. The flow cytometry laboratories involved in this study regularly participated in the external quality control of the CD34+ cell count. Apheresis began on Days 9-12 when the CD34+ count was >10 cells/uL or the WBC count was >2 × 10^6^/L and continued daily for up to 4 days or until more than or equal to 6 × 10^6^ CD34+ cells/kg were collected.

G-CSF+ PXF: Patients received 300 μg/d G-CSF for 4 consecutive days. All patients received a subcutaneous injection of PXF (20 mg or 0.16 mg/kg in case of reduced renal function) at 22:00 on Day 4, regardless of the CD34+ cell count. Apheresis was started at 9:00 on the 5th day. A collection endpoint of at least 2 × 10^6^/kg CD34+ cells sometimes required more than a single harvesting procedure. The target for 2 ASCT was defined as at least 6 × 10^6^/kg CD34+ cells.

### Study endpoints

The primary study endpoint was a comparison of the four mobilization strategies in terms of the percentage of patients achieving the stem cell dose for double ASCT (6×10^6^/kg CD34+). The secondary endpoints were defined as follows: (1) percentage of patients who had a stem cell dose collected ≥2 × 10^6^/kg; (2) total number of stem cells collected and Day 1+Day 2 stem cells collected; (3) rate of on-demand PXF administration; (4) incidence of adverse events occurring during mobilization; (5) factors influencing stem cell mobilization outcomes; and (6) total cost of mobilization and apheresis;(6) incremental cost-effectiveness ratios.

### Costs and cost-effectiveness ratio

All charges and quantities for the mobilizing agents and apheresis were collected from the centralized computer billing system of the hospitals. Only direct medical costs were included in our cost analysis, i.e., costs related to mobilizing drugs, hospitalization, apheresis, CD 34 + cell testing, the cost of the infusion of blood components, and the cost of prophylactic antibiotics. The calculation for the incremental cost effectiveness ratio (ICER) refers to the study of Milone et al. (12).

### Statistical analysis

Categorical variables are presented as counts and percentages, while continuous variables are expressed as mean ± standard deviation. The normality of data distribution was assessed using the Shapiro-Wilk test. For comparisons between two groups: Data following a normal distribution were analyzed using the T-test. The Wilcoxon test was applied to non-normally distributed data. Proportional data were assessed with the chi-square test or Fisher’s exact test, as appropriate. For comparisons among four groups: Univariate ANOVA was employed to evaluate continuous data (like age) differences. When assessing differences across the groups, Bonferroni correction was applied for pairwise comparisons. Proportional data were evaluated with the chi-square test or Fisher’s exact test. For datasets diverging from normal distribution: The Bonferroni approach was utilized for multiple sample rate pairwise comparisons. The Kruskal-Wallis H test was chosen for non-normally distributed data. To calculate adjusted odds ratios and identify potential confounders, significant variables from univariate analyses were incorporated into multivariate logistic regression models. Two distinct stem cell dose thresholds (2 and 6 ×10^6^/kg) served as dependent variables. A significance level of P < 0.05 was used for all statistical tests. All statistical evaluations were conducted using SPSS version 22.0.

## Results

### Patient characteristics

A total of 350 patients met the inclusion criteria of the study, including 66 patients in the Cy+PEG G-CSF+/-PXF group, 91 patients in the Cy+PEG G-CSF group, 169 patients in the Cy+ G-CSF group, and 24 patients in the G-CSF+PXF group. A comprehensive overview of patient demographics is summarized in **Table 1**. The patients in the G-CSF+PXF group were older than those in the other three groups. Other main characteristics remained largely consistent across all study cohorts.

**Table 1:** Baseline characteristics at diagnosis of study population with each mobilization strategy.

Variable	Cy+PEG G-CSF+/-PXF n=66	Cy+PEG G-CSF n=91	G-CSF+PXF n=24	Cy+ G-CSF n=169	P
Age, year, median (IQR)	53 (45,59)	54 (46,60)	64 (58,66)	53 (45,59)	<0.001
Male, n (%)	36 (54.5%)	58 (63.7%)	13 (54%)	102 (60.4%)	0.357
Time from diagnosis to mobilization (mo), median (IQR)	5 (4, 6)	5 (4, 6)	6 (4.8, 6.3)	4 (3, 6)	0.058
ISS, n (%)					0.091
I	17 (25.8%)	20 (22.0%)	5 (20.80%)	63 (37.3%)	
II	35 (53.0%)	51 (56.0%)	13 (54.2%)	62 (36.7%)	
III	14 (21.2%)	20 (22.0%)	6 ( 25.0%)	44 (26.0%)	
Prior radiation therapy, n (%)	1 (1.5%)	1 (1.1%)	0 (0%)	1 (0.6%)	0.468
Disease status before mobilization, n (%)					0.610
CR	21 (31.8%)	35 (38.5%)	8 (33.3%)	58 (34.3%)	
VGPR	35 (53.0%)	42 (46.1%)	14 (58.3%)	79 (46.7%)	
PR	10 (15.2%)	9 (9.9%)	2 (8.4%)	28 (16.6%)	
SD	0 ( 0%)	5 (5.5%)	0 ( 0%)	4 (2.4%)	
Previous lenalidomide exposure, n (%)					0.087
None	56 (84.8%)	69 (75.8%)	19 (79.2%)	150 (88.8%)	
1-4cycles	10 (15.2%)	15 (16.5%)	5 (20.8%)	12 (7.1%)	
≥5 cycles	0 (0%)	7 (7.7%)	0 (0%)	7 (4.1%)	
Prior exposure to alkylating agent, n (%)					0.936
YES	4 ( 6.1%)	5 (5.5%)	1 (4.2%)	12 (7.1%)	
NO	62 (93.9%)	86 (94.5%)	23 (95.8%)	157 (92.9%)	
Percent bone marrow PCs at diagnosis, median (IQR)	26% (13%, 37.5%)	22% (12%, 40.8%)	37.5% (17.8%, 59.9%)	24% (13%, 40.4%)	0.611
Hemoglobin at diagnosis (g/L), median (IQR)	107 (75,124)	94 (79,116)	86 (61.5,116.5)	98 (79.3,116)	0.373
Serum creatinine at diagnosis (umol/L), median (IQR)	78.5 (64, 102.5)	75 (62.5, 138)	70 (49, 131)	85.9 (66, 114)	0.646
Serum albumin at diagnosis (g/L), mean ± standard deviation	36.63 ± 7.28	33.34 ± 7.36	35.89 ± 6.89	36.11 ± 7.31	0.058
Serum calcium at diagnosis (mmol/L), median (IQR)	2.35 (2.26, 2.43 )	2.33 (2.20, 2.50)	2.40 (2.27, 2.43)	2.38 (2.22,2.79 )	0.649
LDH at diagnosis (U/L), median (IQR)	182 (143.95, 215.25)	169 (130, 203)	180 (139, 271)	157 (126, 199)	0.153

ISS, international staging system; CR, complete response; VGPR, very good partial response; PR, partial response; SD, stable disease; PD, progressive disease; LDH, lactate dehydrogenase; Cy, cyclophosphamide; G-CSF,granulocyte colony-stimulating factor; G-CSF,granulocyte colony-stimulating factor; PEG G-CSF, pegylated recombinant human granulocyte colony-stimulating factor; PXF, plerixafor.

### Efficacy of stem cell mobilization and apheresis results

**Figure 1:**
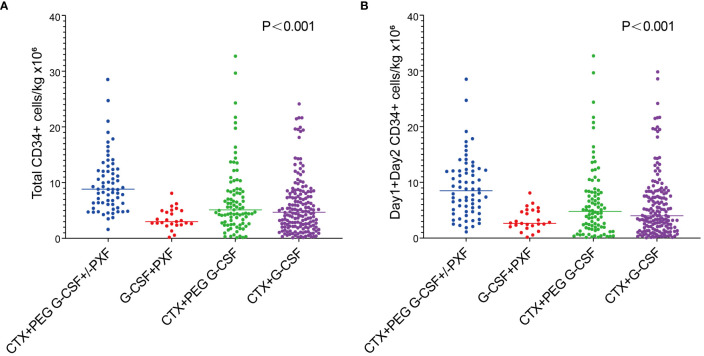
**(A)** Total CD34+ cell yield in each groups. **(B)** Day1+Day 2 CD34+ cell yield in each groups.

The percentage of patients who achieved > 6 × 10^6^/kg CD34^+^ cells, the target dose for double ASCT, was markedly higher in the Cy+PEG G-CSF+/-PXF group (77.3%) than in the other mobilization regimens: Cy+PEG G-CSF (41.8%; P<0.001), Cy+ G-CSF (37.3%; P<0.001), and G-CSF+PXF (8.3%; P<0.001). A harvest of at least 2 ×10^6^/kg CD34+ cell, the minimal target dose for single ASCT, was reached in 98.5% of the Cy+PEG G-CSF+/-PXF group, 84.6% of the Cy+PEG G-CSF group, 77.5% of the Cy+G-CSF group, and 91.7% of the G-CSF+PXF group (P= 0.001).The median aggregate CD34+ cell yield (×10^6^/kg) across the groups was as follows: Cy+PEG G-CSF+/-PXF at 8.79, Cy+PEG G-CSF at 4.96, Cy+G-CSF at 4.65, and G-CSF+PXF at 2.99 (P<0.001) (**Figure 1A**). Assessing the median yields on Day 1 and Day 2 combined, values (×10^6^/kg) were: Cy+PEG G-CSF+/-PXF at 8.41, Cy+PEG G-CSF at 4.62, Cy+G-CSF at 3.92, and G-CSF+PLX at 2.6 (P<0.001) (**Figure 1B**). In our cohort, PXF was deployed for 51.5% of patients in the Cy+PEG G-CSF+/-PXF group with an adaptive strategy and universally for those in the G-CSF+PXF group. On proceeding to ASCT, the average infused CD34^+^ cell count (×10^6^/kg) was PEG G-CSF+/-PXF at 3.31, Cy+PEG G-CSF at 2.74, Cy+G-CSF at 3.17, and G-CSF+PXF at 2.74 (P=0.657). There was no statistical difference in the median time to neutrophil (P=0.324)and platelet engraftment across the groups (P=0.248) (**Table 2**).

**Table 2:** Stem cell mobilization and transplant outcomes with each mobilization strategy.

Variable		Cy+PEG G-CSF+/-PXF n=66	Cy+PEG G-CSF n=91	Cy+G-CSF n=169	G-CSF+PXF n=24	P
Total CD34+ cells collected,×10^6^/kg, median (IQR)		8.79 (6.09, 12.16)	4.96 (2.12, 8.05)	4.65 (2.12,8.05)	2.99 (2.47,4.96)	<0.001
Day1+Day2 CD34+ cellscollected,×10^6^/kg, median (IQR)		8.41 (4.99,12.03)	4.62 (1.88,8.22)	3.92 (1.77,7.98)	2.6 (2.11,4.88)	<0.001
Addition of plerixafor, %		34/66 (51.5%)	0	0	24/24 (100%)	<0.001
No. of patients who achieved >2×10^6^/kg in two days (n,%)		65/66 (98.5%)	77/91 (84.6%)	131/169 (77.5%)	22/24 (91.7%)	0.001
No. of patients who achieved>6×10^6^/kg in two days (n,%)		51/66 (77.3%)	38/91 (41.8%)	63/169 (37.3%)	2/24 (8.3%)	<0.001
Infused CD34+/kg CD34+cells thansfused, median (range)		3.31 (2.27, 4.52)	2.74 (2.24, 4.66)	3.17 (2.11, 4.90)	2.74 (2.51, 3.16)	0.657
Days to neutrophil engraftment, median (range)		10 (9,11)	10 (9,10)	11 (9,11)	10 (10,11)	0.324
Days to platelets engraftment, median (range)		11 (10,13)	12 (11,13)	14 (13,15)	12 (10, 14)	0.248
All AEs, N (%)	Total AEs	26 (39.4%)	44 (48.4%)	4 (16.7%)	82 (48.5%)	0.021
	none	40 (60.7%)	47 (51.6%)	20 (83.3%)	87 (51.5%)	
	one AE	25 (37.8%)	39 (42.9%)	3 (12.5%)	60 (35.5%)	
	≥one AE	1 (1.5%)	5 (5.5%)	1 (4.2%)	22 (13.0%)	
Nausea, N (%)		18 (27.3%)	23 (25.3%)	1 (4.2%)	49 (29.0%)	0.072
	Grade 1-2	16 (24.2%)	22 (24.2%)	1 (4.2%)	47 (27.8%)	
	Grade 3-5	2 (3.3%)	1 (1.1%)	0	2 (1.2%)	
diarrhea		6 (9.1%)	9 (9/9%)	4 (16.7%)	16 (9.5%)	0.752
	Grade 1-2	6 (9.1%)	8 (8.8%)	4 (16.7%)	16 (9.5%)	
	Grade 3-5	0	1 (1.1%)	0	0	
Neutropenic fever, N (%)		2 (3.0%)	9 (9.9%)	0	29 (17.2%)	0.008
Transfusions, N (%)		18 (27.3%)	28 (30.8%)	0	37 (21.9%)	0.005
	Red blood cells	1 (1.5%)	6 (6.6%)	0	6 (3.6%)	0.276
	Platelets	18 (27.3%)	25 (27.5%)	0	35 (20.7%)	0.015
Cystitis, N (%)		1 (1.5%)	0	0	1 (0.6%)	0.670

Cy, cyclophosphamide; G-CSF,granulocyte colony-stimulating factor; PEG G-CSF, pegylated recombinant human granulocyte colony-stimulating factor; PXF, plerixafor; AEs, adverse events.

### Factors influencing stem cell mobilization outcomes

The multivariate analysis results are depicted in **Table 3**. Statistically significant variables from the univariate analysis were gathered to establish multivariate models, considering two distinct stem cell dose thresholds as dependent variables: 2×10^6^/kg and 6×10^6^/kg. Patients who underwent lenalidomide treatment for either ≥ 5 cycles (OR: 0.099; 95% CI: 0.030-0.322;P<0.001) or ≤ 4 cycles (OR: 0.265; 95% CI: 0.112-0.630; P=0.003) were less likely to achieve the 2×10^6^ CD34^+^ cells/kg, compared to those without any lenalidomide exposure. Having a disease status of ≥ VGPR before mobilization, as opposed to < VGPR, increased the chances of reaching the target of 2×10^6^ CD34^+^ cells/kg (OR: 2.434; 95% CI: 1.163-5.092; P=0.018). The Cy+PEG G-CSF+/-PXF mobilization strategy demonstrated a higher success rate in achieving the 2×10^6^ CD34^+^ cells/kg target than the Cy+G-CSF approach. Yet, no significant difference was identified between the Cy+PEG G-CSF+/-PXF and G-CSF+PXF regimens. Regarding the higher threshold of 6×10^6^ CD34^+^ cells/kg: A pre-mobilization disease status of ≥ VGPR increased the probability of success (OR: 1.918; 95% CI: 1.004-3.663; P=0.049), as did the utilization of Cy + PEG G-CSF +/-PXF for mobilization. Patients aged between 65 and 70 were less likely to achieve this benchmark when compared to those aged < 65 (OR: 0.234; 95% CI: 0.073-0.748; P=0.014). Further, a longer lenalidomide treatment duration (≥ 5 cycles) diminished the likelihood of reaching this goal when compared to no treatment (OR: 0.099; 95% CI: 0.012-0.781; P=0.028). Interestingly, the difference between no lenalidomide exposure and exposure for ≤ 4 cycles was not statistically significant for this threshold.

**Table 3:** Multivariate analysis (Logistic Regression) of factors influencing mobilization outcomes.

Variable	Odds Ratio		95% CI	P
Total CD34+ cells collected 2×10^6^/kg				
Lenalidomide exposure				
≤ 4 cycles vs. None	0.265		0.112-0.630	0.003
≥ 5cycles vs. None	0.099		0.030-0.322	<0.001
Disease status before mobilization				
≥ VGPR vs. <VGPR	2.434		1.163-5.092	0.018
Mobilization regimen				
Cy+PEG G-CSF+/-PXF vs.Cy +G-CSF	17.461		2.302-132.452	0.006
Cy + PEG G-CSF +/-PXF vs. Cy +PEG G-CSF	8.308		1.036-66.645	0.046
Cy + PEG G-CSF +/-PXF vs. G-CSF+PXF	4.386		0.367-52.461	0.243
Total CD34+ cells collected 6×10^6^/kg				
Lenalidomide exposure				
≤ 4 cycles vs. None	0.734		0.344-1.566	0.424
≥ 5cycles vs. None	0.099		0.012-0.781	0.028
Disease status before mobilization				
≥ VGPR vs. <VGPR	1.918		1.004-3.663	0.049
Mobilization regimen				
Cy+PEG G-CSF+/-PXF vs.Cy +G-CSF	5.350		2.665-10.741	<0.001
Cy + PEG G-CSF +/-PXF vs.Cy +PEG G-CSF	3.993		1.872-8.513	<0.001
Cy + PEG G-CSF +/-PXF vs. G-CSF+PXF	23.611		4.705-118.491	<0.001
Age				
65-70 vs. < 65	0.234		0.073-0.748	0.014

VGPR, very good partial response; Cy, cyclophosphamide; G-CSF, granulocyte colony-stimulating factor; PEG G-CSF, pegylated recombinant human granulocyte colony-stimulating factor; PXF, plerixafor.

### Adverse events

Within our cohort, AEs were observed in different frequencies among the treatment groups: 39.4% (26 patients) in the Cy + PEG G-CSF +/-PXF group, 48.4% (44 patients) in the Cy + PEG G-CSF group, 48.52% (82 patients) in the Cy +G-CSF group, and 16.67% (4 patients) in the G-CSF+PXF group. The predominant AEs encountered were nausea and diarrhea, with most being of mild intensity. The majority of AEs were categorized as grades 1-2 based on the Common Terminology Criteria for Adverse Events (CTCAE) version 4.0. Instances of neutropenic fever varied among groups: 3.0% in the Cy + PEG G-CSF +/-PXF group, 9.9% in the Cy + PEG G-CSF group, and 17.2% in the Cy + G-CSF group. Importantly, no severe (grade 4 or 5) infections were identified (**Table 2**). Red blood cell transfusions were administered when hemoglobin levels dropped below 60 g/L, and platelet transfusions were provided when platelet counts descended below 20×10^9^/µl. Transfusion requirements were as follows: Red blood cell transfusions were necessary for 1.5% of patients in the Cy + PEG G-CSF +/-PXF group, 6.6% in the Cy + PEG G-CSF group, and 3.6% in the Cy + G-CSF group. For platelet transfusions, 27.3% of patients in the Cy + PEG G-CSF +/-PXF group, 27.5% in the Cy + PEG G-CSF group, and 20.7% in the Cy + G-CSF group required the intervention. Hemorrhagic cystitis was a rare complication, appearing in only 1.5% of patients in the Cy + PEG G-CSF (+/-PXF) group and 0.6% in the Cy + G-CSF group. In affected patients, the condition resolved in under 48 hours with adequate hydration and bladder irrigation.

### Financial analysis and incremental cost-effectiveness ratios

**Figure 2:**
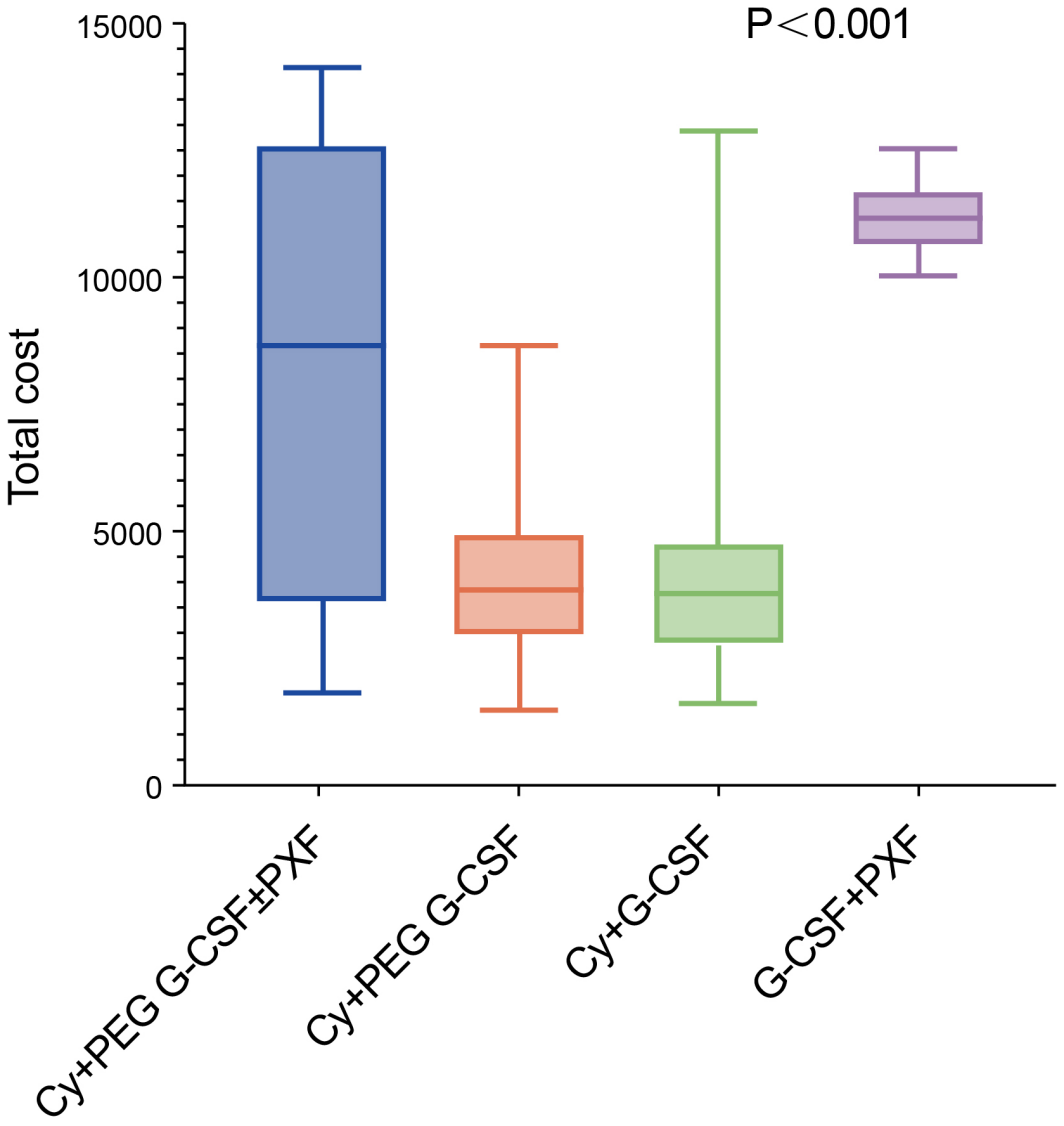
The total cost of mobilization and apheresis using in each groups.

The total cost of mobilization and apheresis using Cy +PEG G-CSF +/-PXF was significantly lower at $ 5,929.52 (1,727.07 -11,711.29)compared to the G-CSF+PXF group at $11,146.86

(10,563.68-11,195.07) and significantly higher than the Cy+ PEG G-CSF group at $ 2,485.31 (1,959.00-2,991.23) and the Cy+ G-CSF group at $ 1,880.81(1,637.50 -2,474.67) (P< 0.001) (**Figure 2**). From an economic perspective, when considering the enhancement in success rates, the Cy + PEG G-CSF +/-PXF group had an incremental cost-effectiveness ratio (ICER) of $247.78 for each 1% rise in achieving the minimum threshold of 2×10^6^ CD34^+^ cell apheresis harvest (designated as ICER-1). Furthermore, for attaining the higher benchmark of 6×10^6^ CD34^+^ cell harvest, the ICER stood at $97.02 per 1% increase (ICER-2) (**Table 4**).

**Table 4:** Incremental cost-effectiveness ratio (ICER) of the on-demand PXF group compared to the control group.

Variable	Formula	Data	Results incremental cost-effectiveness ratio (ICER)
ICER-1 (successful minimal harvest) ≥2×10^6^ D34+ cells/kg	(cost of mobilization in Cy+PEG G-CSF+/-PXF study group - cost of mobilization in Cy+PEG G-CSF group)/(% of patients achieving minimal harvest in Cy+PEG G-CSF+/-PXF study group -% of patients achieving minimal harvest in Cy+PEG G-CSF group)	(5929.52-2,485.31)÷ (98.5-84.6)=3,444.21÷13.9 = 247.78	247.78
ICER-2 (successful optimal harvest) ≥6 ×10^6^ D34+ cells/kg	(cost of mobilization in Cy+PEG G-CSF+/-PXF study group - cost of mobilization in Cy+PEG G-CSF group)/(% of patients achieving optimal harvest in Cy+PEG G-CSF+/-PXF study group -% of patients achieving optimal harvest in Cy+PEG G-CSF group)	(5929.52-2,485.31)÷(77.3-41.80)= 3,444.21÷35.5 = 97.02	97.02

ICER, incremental cost-effectiveness ratio; Cy, cyclophosphamide; PEG G-CSF, pegylated recombinant human granulocyte colony-stimulating factor; PXF, plerixafor.

## Discussion

While newer therapeutic agents have emerged, offering profound responses, ASCT continues to be the cornerstone for eligible newly diagnosed MM patients. Securing two or more sufficient stem cell grafts ensures the possibility of tandem ASCT or rescue ASCT for appropriate MM patients. Historically, two primary stem cell mobilization techniques prevailed: utilizing growth factors like G-CSF by itself or combined with chemotherapy. However, these techniques do not uniformly benefit all patients (12, 28).

The integration of high-dose cyclophosphamide (Cy) (3 g/m^2^) with G-CSF, supplemented by PXF as needed, has gained traction as an effective mobilization strategy. Yet, the G-CSF regimen demands daily, multiple injections. On the other hand, PEG G-CSF requires only a one-time injection at a 6 mg dosage, given its prolonged serum duration. This makes PEG G-CSF not only more user-friendly but also reduces discomfort.

In this study, the mobilizing strategy of Cy+PEG G-CSF+/-PXF obtained results statistically superior to those obtained using Cy+ PEG G-CSF, Cy+G-CSF, and G-CSF+PXF. To the best of our knowledge, this is the first study to compare Cy+PEG G-CSF +/-PXF with other mobilization schemes. Our data suggests that adding on-demand PXF to Cy and PEG G-CSF improved mobilization outcomes in comparison to the control groups (Cy+ PEG G-CSF and Cy+G-CSF). A striking 77.3% of patients in the Cy+PEG G-CSF+/-PXF group achieved the benchmark of >6×10^6^/kg—sufficient for two transplants—manifestly surpassing the Cy+PEG G-CSF (41.8%; P<0.001) and Cy+G-CSF groups (37.3%; P<0.001). Furthermore, these commendable outcomes necessitated limited PXF interventions (only 51.5%), contributing to an impressive cost-effectiveness ratio. Notably, our data discerned the superiority of Cy+PEG G-CSF+/-PXF over G-CSF+PXF. Prior research indicated a substantial failure rate with G-CSF monotherapy mobilization (9, 10). Echoing these findings, and we also noted a high rate of mobilization failure in this group. Consequently, we integrated PXF as a standard measure, ensuring its administration to all patients receiving G-CSF, irrespective of their precollection stem cell count. Our study found that even if all patients in the G-CSF group received PXF, the mobilization effect of Cy+PEG G-CSF+/-PXF was still better than that of G-CSF+PXF. It is imperative to acknowledge the age discrepancy across our study groups. The cohort undergoing G-CSF+ PXF predominantly comprised elderly participants, while the remaining groups were relatively younger. Recognized determinants, such as age (29, 30), prior lenalidomide exposure (31), and pre-mobilization disease status (32), undeniably influence mobilization and harvest outcomes in MM. Hence, the pronounced efficacy of Cy+PEG G-CSF+/-PXF might be attributed, at least partially, to the younger age profile in this group compared to the G-CSF+PXF patients. However, our multivariable logistic regression analysis further validated the superior HSC mobilization and harvest outcomes of the Cy+PEG G-CSF+/-PXF cohort. In this analysis, the differences in age, lenalidomide exposure, and disease status before mobilization were taken into account. It highlighted that even when every patient in the G-CSF+PXF group was consistently treated with PXF, the mobilization efficacy of Cy+PEG G-CSF+/-PXF remained superior to G-CSF+PXF. A significant consideration was the financial implications of universally administering PXF, given that it is administered to all patients in the G-CSF+PXF group. Our findings indicated a higher percentage of patients achieving the desired stem cell yield (≥6 × 10^6^ CD34^+^ cells/kg, suitable for tandem transplantation) in the Cy+PEG G-CSF+/-PXF cohort compared to G-CSF+PXF as documented in earlier studies (77% vs. 72%) (10).

Currently, many experts advocate for a risk-adjusted approach, incorporating PXF “on-demand”, as the mainstream protocol for stem cell mobilization. The synergy of high-dose cyclophosphamide (Cy) (3 g/m^2^), PEG G-CSF, and on-demand PXF in our investigation aligns closely with outcomes from the CTX+G-CSF+/-PXF arm in previous randomized trials. A retrospective study by Beatrice et al. evaluated HSC mobilization in 422 MM patients. Their cohorts included 188 patients administered with low-dose Cy (LD-Cy, defined as 2 g/m^2^), 163 with intermediate to high-dose Cy (ID-Cy, designated as 3 g/m^2^), and 71 patients with G-CSF monotherapy. Although they did not specify the percentage reaching >6×10^6^/kg CD34^+^ cells, they reported an impressive 90.4% in the LD-Cy and 91.1% in the ID-Cy groups achieving a minimum HSC dose of 4×10^6^ (20). In a retrospective analysis, Andrew et al. assessed 398 patients mobilized with either Cy (4 g/m^2^) combined with G-CSF or G-CSF as a standalone therapy. Both groups had PXF introduced on-demand. Notably, 94% of the participants in their study achieved the desired yield of 4×10^6^ CD34^+^/kg (19). Similarly, Giuseppe Milone and his team evaluated 111 MM patients undergoing mobilization with Cy at 4 g/m^2^ and G-CSF, revealing that 84.6% reached the >4×10^6^/kg threshold (12). In our research, utilizing Cy plus PEG G-CSF in conjunction with on-demand PXF, a remarkable 93.4% met the same >4×10^6^/kg criterion. However, we should note the variations across these studies, both in the target stem cell collection thresholds and the respective algorithms employed for on-demand PXF. Different algorithms might influence the proportion of patients necessitating PXF. However, the percentage of patients in our study who needed PXF (51.5%) was higher than the percentage of patients in previous studies who needed PXF (12.3%-28%) (18, 20, 21). However, previous studies have used multiple injections of PXF until the target stem cells are collected. In our study, PXF was given as a single dose at a fixed dose. This inherent difference makes direct comparisons between mobilization outcomes and the economic ramifications of Cy+PEG G-CSF+/-PXF and Cy+G-CSF +/- PXF a challenge. A subsequent analysis did endorse the efficacy of a solitary, pre-set PXF dose for stem cell mobilization. Significantly, more patients proceeded with successful ASCT post-PXF administration: a leap from 59.6% pre-plerixafor to 90% post-plerixafor (P<0.001) (33). The financial upside of this approach is evident. By restricting the drug’s administration to a single on-demand, fixed-dose, we mitigated ancillary costs, circumvented potential additional apheresis sessions, and minimized the likelihood of secondary mobilization treatments. The financial analysis revealed that the mobilization and apheresis costs associated with the Cy+PEG G-CSF+/-PXF regimen were markedly less than those of the G-CSF+PXF approach. Since 100% of patients received PXF in the G-CSF+PXF group, when PXF was added to mobilization with G-CSF only, the increase in costs related to the use of PXF was $8,517 per patient. Our study also found that the total cost of mobilization and apheresis using Cy+PEG G-CSF+/-PXF was significantly higher than that of the Cy+PEG G-CSF group. In our assessment, the additional expenditure linked to PXF’s integration was $3,444 per patient – a figure that remains financially reasonable. The incremental cost-effectiveness ratio for each 1% increase in the probability of achieving a successful optimal harvest (>6×10^6^/kg) was $97.02 per patient. In the context of the on-demand PXF regimen, while the initial mobilization does carry a steeper price due to PXF’s inclusion, subsequent “rescue” mobilizations are more economical. Thus, when balancing out initial and follow-up mobilization expenses, the on-demand approach proves cost-efficient overall.

Chemotherapy-related mobilization raises legitimate toxicity concerns. Yet, in our study, there were no instances of treatment-induced fatalities prior to ASCT. Overall, the side effects linked to the Cy+PEG G-CSF+/-PXF regimen were controllable. The most frequently reported AEs were nausea and vomiting, with the vast majority being mild. The bulk of these AEs were classified as grades 1-2. Furthermore, only a scant 3.0% of participants experienced neutropenic fever, which, in most instances, was of short duration. Only one case of hemorrhagic cystitis was reported in the patients treated with cyclophosphamide (1.5%) but resolved quickly with a less than 48-hour admission.

Given our findings, we advocate for the application of Cy+PEG G-CSF+/-PXF for those patients in which tandem transplantation is planned or if ASCT at relapse is considered. On the other hand, In contrast, in more fragile patients or if a single ASCT is recommended for age, the G-CSF+PXF combination seems to be a more suitable alternative. Our research underscores that for MM patients earmarked for ASCT, incorporating on-demand PXF with HD-Cy and PEG G-CSF not only ensures a robust stem cell yield but is also a cost-effective and safe mobilization approach.

## Data availability statement

The raw data supporting the conclusions of this article will be made available by the authors, without undue reservation.

## Ethics statement

The studies involving humans were approved by Clinical Research and Animal Ethics Committee of The First Affiliated Hospital of Sun Yat-sen University. The studies were conducted in accordance with the local legislation and institutional requirements. Written informed consent for participation was not required from the participants or the participants’ legal guardians/next of kin in accordance with the national legislation and institutional requirements.

## Author contributions

L-QH: Writing – original draft. J-RL: Writing – review & editing. J-LG: Formal analysis, Methodology, Writing – review & editing. M-LC: Writing – review & editing. L-FK: Writing – review & editing. B-HH: Writing – original draft. W-YZ: Writing – original draft. JL: Conceptualization, Methodology, Resources, Supervision, Visualization, Writing – original draft.
